# The role of melatonin in the onset and progression of type 3 diabetes

**DOI:** 10.1186/s13041-017-0315-x

**Published:** 2017-08-01

**Authors:** Juhyun Song, Daniel J. Whitcomb, Byeong C. Kim

**Affiliations:** 1 Department of Biomedical Sciences, Center for Creative Biomedical Scientists at Chonnam National University, Gwangju, 61469 South Korea; 2 Henry Wellcome Laboratories for Integrative Neuroscience and Endocrinology, School of Clinical Sciences, Faculty of Healthy Sciences, University of Bristol, Whitson street, Bristol, BS1 3NY UK; 3 Department of Neurology, Chonnam National University Medical School, Gwangju, 61469 South Korea

**Keywords:** Melatonin, Type 3 diabetes, Alzheimer’s disease (AD), Insulin resistance, Hyperglycemia, Blood brain barrier (BBB), Beta amyloid (Aβ)

## Abstract

Alzheimer’s disease (AD) is defined by the excessive accumulation of toxic peptides, such as beta amyloid (Aβ) plaques and intracellular neurofibrillary tangles (NFT). The risk factors associated with AD include genetic mutations, aging, insulin resistance, and oxidative stress. To date, several studies that have demonstrated an association between AD and diabetes have revealed that the common risk factors include insulin resistance, sleep disturbances, blood brain barrier (BBB) disruption, and altered glucose homeostasis. Many researchers have discovered that there are mechanisms common to both diabetes and AD. AD that results from insulin resistance in the brain is termed “type 3 diabetes”. Melatonin synthesized by the pineal gland is known to contribute to circadian rhythms, insulin resistance, protection of the BBB, and cell survival mechanisms. Here, we review the relationship between melatonin and type 3 diabetes, and suggest that melatonin might regulate the risk factors for type 3 diabetes. We suggest that melatonin is crucial for attenuating the onset of type 3 diabetes by intervening in Aβ accumulation, insulin resistance, glucose metabolism, and BBB permeability.

## Introduction

Alzheimer’s disease (AD) is an age-related neurodegenerative disorder that is characterized by the abnormal aggregation and accumulation of toxic peptides resulting in beta amyloid (Aβ) plaques and intracellular neurofibrillary tangles (NFT) [1]. According to recent reports, the number of patients with AD will be over 13.8 million by 2050, which will place a tremendous burden on society globally [2–4]. The onset of AD is linked to various causes, such as genetic mutations [5, 6], sex [7], lipid metabolism [8–11], aging [12–14], and diet [9, 15]. AD pathology results from excessive oxidative stress, synaptic loss, neuronal cell death, impaired insulin signaling, and abnormal glucose metabolism [16–18]. Cohort studies have demonstrated that type 2 diabetes (T2DM) increases the risk of dementia and results from common risk factors associated with dementia, including insulin resistance and hyperglycemia [19]. Many patients with metabolic diseases, such as cardiovascular disease, diabetes, and obesity, are reported to have a progressive decline in cognitive function, leading to the development of AD [20, 21]. One meta-analysis showed that diabetes significantly increases the risk for AD in elderly people [22]. Owing to the common risk factors between diabetes and AD, recent studies have proposed that AD is a brain-specific type of diabetes, which they termed “type 3 diabetes” [17, 23–25].

Melatonin (N-acetyl-5-methoxytryptamine) is mainly secreted as a neurohormone by the pineal gland [26]. It plays a role in various physiological functions, including circadian rhythm regulation, antioxidant activities, and the regulation of mitochondrial function [27–30]. Given that sleep disorders frequently occur in up to 45% of patients with AD [31–33], melatonin is an important hormone for the treatment of AD since it corrects abnormal sleep patterns [34, 35]. In AD, melatonin levels are decreased in the cerebrospinal fluid (CSF) compared to those in the normal population [36, 37]. Several studies have demonstrated that melatonin reduces Aβ accumulation [38], tau hyperphosphorylation [39], synaptic dysfunction [40], and blood brain barrier (BBB) permeability [41]. Moreover, melatonin attenuates insulin resistance [42], and regulates glucose homeostasis [43, 44]. In this review, we summarize the therapeutic functions of melatonin in type 3 diabetes from various perspectives.

### The risk factors for diabetes contribute to the onset and progression of Alzheimer’s disease

#### Insulin resistance leads to cognitive decline

**Fig. 1:**
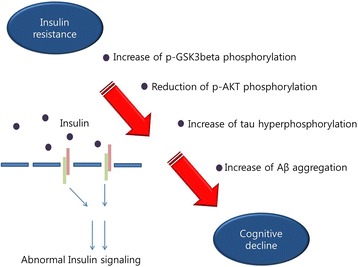
Insulin resistance triggers cognitive decline. Insulin resistance increases p-GSK3β phosphorylation, tau hyperphosphorylation, Aβ aggregation and reduces p-AKT phosphorylation, leading to cognitive deficits

Diabetes is characterized by insulin resistance, diminished pancreatic beta-cell function, and abnormally high glucose levels [45]. Diabetes is commonly classified into two types, namely, type 1 (T1DM) and T2DM [45]. T2DM occurs more frequently in the global population than T1DM and is accompanied by insulin resistance, hyperglycemia, cognitive decline, and impaired circadian rhythms [46, 47]. T2DM is known to be associated with cognitive impairments [48], and is commonly used as an index for the development of vascular dementia [49], and AD [50, 51]. The high prevalence of central nervous system (CNS) diseases in patients with diabetes has already been revealed by global reports [52–55]. The onset and progression of AD is associated with the capacity of the brain to utilize glucose for energy production [56, 57]. In the CNS, insulin signaling plays central roles in the cognitive dysfunction found in AD [58]. Insulin is known to be neuroprotective and has powerful effects on memory [59]. Previous studies have shown that deficiencies in insulin receptors (IRs) in the brain, a factor implicated in insulin resistance, leads to memory dysfunction [18, 60]. IRs are localized in cerebral regions, such as the hippocampus, amygdala, and septum [61, 62]. AD patients show an 80% reduction in IRs in their brains compared to normal subjects [17]. Consequently, insulin signaling is abnormal [63]. Some studies have demonstrated that the hippocampus regulates the consolidation of memory via insulin signaling [64, 65]. Based on this evidence, decreased insulin levels were subsequently found in the CSF of patients with AD and mild cognitive impairment (MCI) [60, 66, 67]. Aβ accumulation, abnormalities in the cholinergic system, tau hyperphosphorylation, and damage to neuronal cells contributes to impaired insulin signaling [68, 69]. Insulin receptors deficiency in the AD brain results in insulin resistance in AD neuropathology [18, 70]. For these reasons, reduced levels of insulin receptor genes may contribute to the progression of AD [23, 71]. Moreover, tau pathology in AD is mediated by impaired tau gene expression owing to the attenuation in insulin signaling [72, 73]. Insulin resistance in the AD brain reduces the phosphorylation of phosphoinositol-3-kinase (PI3K), and Akt [72, 73], which ordinarily function to promote neuronal growth and survival, and promotes GABAergic transmission involved in learning and memory [74], and blocks the accumulation of Aβ [75]. Additionally, insulin resistance increases the activation of glycogen synthase kinase (GSK-3) [76, 77], which is related to the hyperphosphorylation of tau and the acceleration of tau misfolding [78]. Indeed, owing to deficiencies in insulin, the change of GSK-3 activity leads to the hyperphosphorylation of tau [79], perhaps unsurprising given what we know about the aberrant activation of GSK-3β and the resultant Aβ accumulation and tau protein phosphorylation [80, 81]. Moreover, several clinical studies have demonstrated a positive correlation between diabetes and AD [57, 82], and suggested that the central reasons for this include aberrant insulin signaling and dementia [58, 83–85]. In in vivo studies, an AD mouse model showed insulin resistance [24], reduced glucose metabolism, oxidative stress, and cognitive impairments [86] following injections of streptozotocin (STZ). In addition, insulin resistance leads to hippocampal neuronal loss owing to amyloid neurotoxicity [68], reduced glucose uptake by inhibiting the expression of glucose transporters in cell membrane [87], and accelerated amyloid aggregation during early AD [88]. Consequently, insulin resistance and impaired insulin signaling are significantly related to tau hyperphosphorylation and Aβ deposition in AD, and ultimately contribute to cognitive decline [69] (Fig. 1).

#### Hyperglycemia triggers BBB disruption leading to cognitive dysfunction

**Fig. 2:**
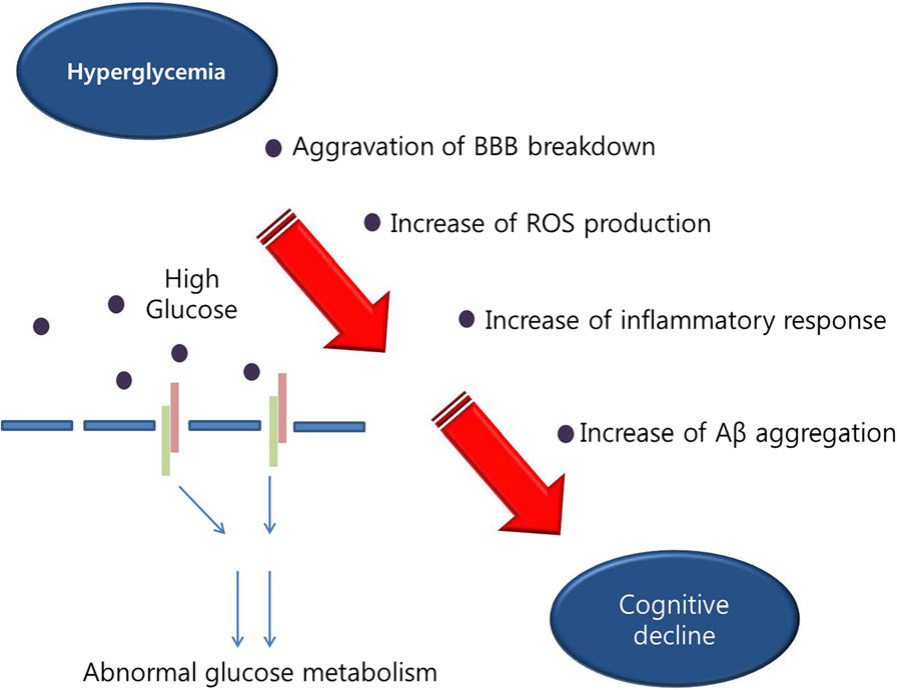
Hyperglycemia leads to cognitive decline. Hyperglycemia aggravate BBB breakdown, and increase the generation of ROS, inflammatory response, and Aβ aggregation, ultimately leading to cognitive decline

According to previous studies, hyperglycemia in T2DM leads to cognitive dysfunction [89–91]. An abnormal glycemic condition is one of the main causes of BBB breakdown in patients with diabetes [92, 93]. Several studies demonstrated that the loss of tight junction proteins which make up the BBB and the activation of matrix metalloproteinases (MMPs) was shown in hyperglycemia in vivo model [94] and in patients [95, 96]. The BBB is comprised of brain endothelial cells lining the cerebral microvessels with astrocytic end-feet processes. The BBB endothelium is characterized by specific transmembrane transport systems that control the trafficking of small molecules in and out of the brain parenchyma [97]. Glucose, the primary energetic source in the brain, can cross the BBB through transporter proteins, such as facilitative sodium independent transporters (e.g., the glucose transporter [GLUT]) [98, 99]. One animal study has shown downregulation of BBB glucose transporters in hyperglycemic mice compared to wild-type mice [100]. In chronic hyperglycemia conditions, GLUT1 and GLUT3 expression was attenuated in diabetic animal brain and subsequently aberrant GLUT’s expression triggers neuronal cell damage [100]. In addition, many studies have reported that the BBB in the diabetic brain has increased permeability owing to the activation of hypoxia-inducible factor-1α (HIF-1α) and increased levels of vascular endothelial growth factor (VEGF) [101, 102]. Hyperglycemia promotes the production of reactive oxygen species (ROS) [103, 104] and downregulates glucose transporters in brain endothelial cells [105]. Moreover, hyperglycemia aggravates amyloid toxicity, independent of insulin resistance [106]. Numerous studies have demonstrated that diet-induced hyperglycemia triggers an increase in BBB permeability and BBB damage [107]. The expression of IgG as the marker of BBB permeability was increased and tight junction proteins were attenuated in a hyperglycemia model [107]. In AD, BBB disruption promotes tau hyperphosphorylation [108, 109]. BBB disruption decreases the expression of glucose transporters [110], promotes ROS production [111] and increases infiltration of inflammatory mediators [112]. Tau aggregation is associated with increase of inflammation [112] and reduction of glucose transporters [110]. In addition, BBB dysfunction in AD contributes to Aβ clearance, activates glial cells, and aggravates inflammation by recruiting leukocytes to the brain [113]. Given this evidence, hyperglycemia-induced BBB disruption might play an important role in the onset and progression of AD (Fig. 2).

### Melatonin in AD

Melatonin has been shown to have neuroprotective effects in a mouse model of AD [114, 115], since it attenuates Aβ accumulation and synaptic dysfunction by stabilizing the mitochondria function and inhibiting DNA damage [38, 40]. Melatonin controls several molecular signaling pathways, such as PI3/Akt/GSk3β and hemooxygenase-1 [39, 116, 117], and free radical scavenging mechanisms [118, 119] in the AD brain. A recent study demonstrated that melatonin improves synapse dysfunction via the Notch1/Hes1 signaling pathway in the hippocampus [120]. Another study suggested that melatonin inhibits apoptotic mediators and promotes pro-survival signaling in a model of AD [121]. An animal study demonstrated that chronic melatonin treatment for 30 days improves memory impairments in the AD mouse model [117]. Moreover, in patients with AD, melatonin levels were significantly decreased in the serum and CSF, and levels of melatonin were considered as a candidate risk factor for diagnosis of AD [37, 122]. Clinically, melatonin and its agonist have been regarded as treatments for AD [123, 124]. As mentioned above, melatonin has the potential to attenuate AD pathology via numerous mechanisms including PI3K/Akt/GSK3β [37] and Notch1 signaling [120], and RAGE/NF-κB/JNK signaling pathway [117]. Future study of the specific mechanisms of melatonin in the CNS is necessary to identify potential therapeutic solutions for AD.

### The relationship between melatonin and type 3 diabetes

#### Melatonin protects cells against Aβ toxicity and inhibits tau hyperphosphorylation

Aβ, the main component of amyloid plaques, is believed to cause memory dysfunction [125]. Melatonin improves soluble Aβ-induced memory dysfunction and synaptic dysfunction via the Musashi1/Notch1/Hes1 signaling pathway [120], suggesting that the modulation of Notch1 could restore neurogenesis and cognitive function in AD models [126]. According to the results of an in vivo study, melatonin administration inhibits the expression of amyloid precursor protein-cleaving secretases in the hippocampus [127]. In addition, melatonin attenuates the memory impairments induced by Aβ accumulation in a sporadic AD model [38, 128, 129]. Melatonin inhibits the transcription of β-secretases via the melatonin receptors in SH-SY5Y neuronal cells [130]. Melatonin attenuates Aβ-induced memory dysfunction and tau hyperphosphorylation via the PI3/Akt/GSK3β pathway in the mouse brain [39]. Melatonin suppresses the activity of GSK3β through activation of p-GSK3β (Ser9) in Aβ in vitro model [131]. Moreover, it improves Aβ-induced impairments in hippocampal long-term potentiation (LTP) in rats [132]. Melatonin inhibits superoxide anion production in microglia under conditions of Aβ toxicity [115]. In addition, it inhibits memory dysfunction and tau phosphorylation in rats [133]. Considering the effect of melatonin on Aβ toxicity and tau hyperphosphorylation in AD, melatonin may be a key to improving memory function by suppressing the cell damage induced by Aβ toxicity and tau hyperphosphorylation.

#### Melatonin protects cells against insulin resistance and hyperglycemia

**Fig. 3:**
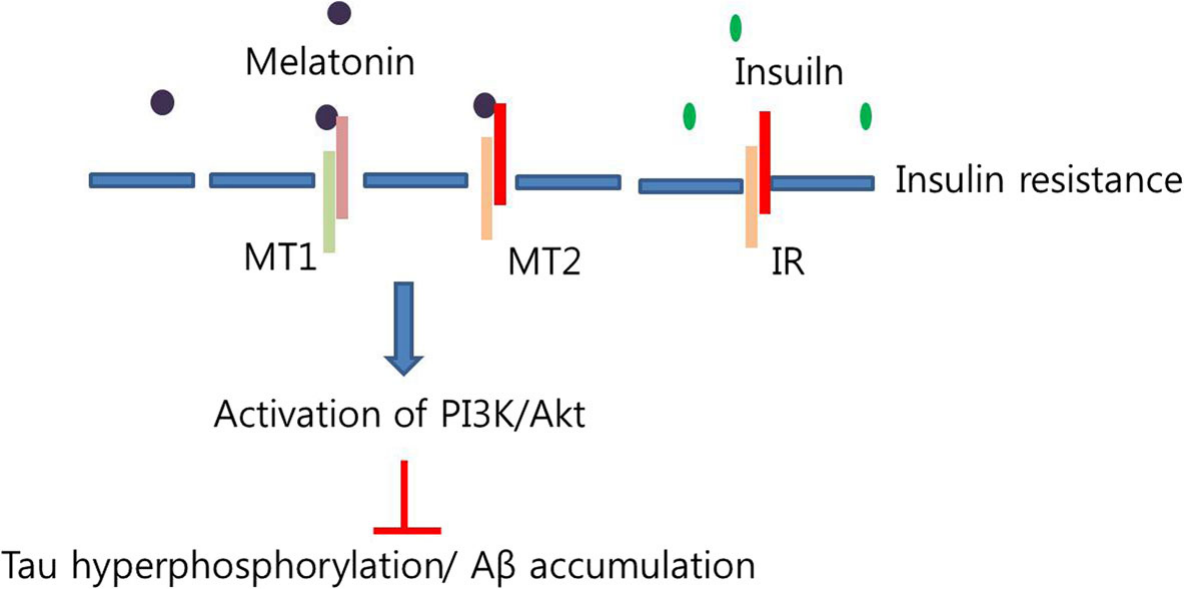
Melatonin restores the disruption of insulin signaling in AD. In insulin resistance condition, melatonin activates PI3K/Akt signaling, leading to the decrease of tau hyperphosphorylation and Aβ accumulation

**Fig. 4:**
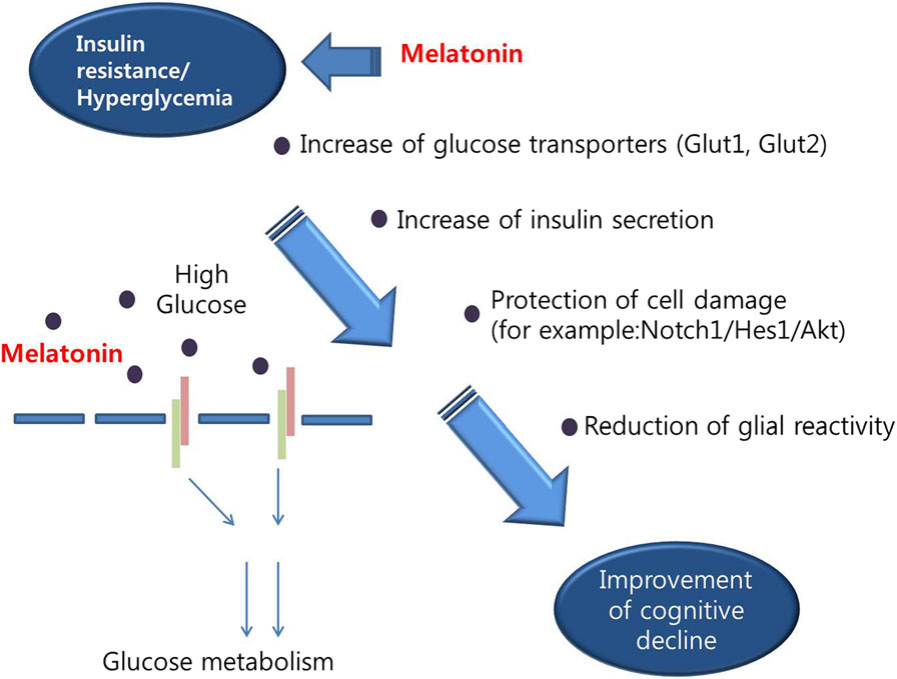
Melatonin improve cognitive decline by regulating insulin resistance and hyperglycemia. Melatonin controls insulin resistance and glucose metabolism. Melatonin increases the expression of glucose transporters such as Glut1 and Glut2. Also, melatonin increase the secretion of insulin and protects cell damage, and reduces glial reactivity. Finally, melatonin improves cognitive decline in AD brain

Diabetes is accompanied by dysregulation of the circadian system [134]. This is interesting given that glucose metabolism is regulated by the circadian system [135, 136]. In animals and humans with diabetes, increased insulin levels and abnormal glucose metabolism triggers aberrant circadian rhythms [42, 137]. One study demonstrated that a reduction of melatonin levels in serum is linked with high insulin levels in T2DM rats [42]. Moreover, Sakotnik et al. suggested that polymorphisms in the melatonin receptor gene are related to fasting blood glucose levels and increases in the prevalence of T2DM [138]. Several genome wide studies have shown that specific single nucleotide polymorphisms of the melatonin receptor 2 (MTNR1B) locus are related to the high glucose levels found in T2DM [139–141]. Genome-wide studies have shown that allelic variations in the melatonin receptor 2 (MT2) contribute to the elevations in fasting glucose levels in plasma, insulin resistance, and ultimately the risk for type 2 diabetes [142, 143]. Type 3 diabetes is related to the prevalence of T2DM and results from insulin resistance and hyperglycemia [144–146]. Therefore, a method of reducing the cell damage induced by insulin resistance and hyperglycemia is crucial in both diabetes and AD. Melatonin activates the expression of the MT2 receptor, which can inhibit the secretion of insulin from pancreatic β-cells [147, 148]. Numerous studies have shown that melatonin contributes to glucose homeostasis and that low glucose levels are present in patients with T2DM [137, 149]. A recent study has shown that loss of the melatonin receptor contributes to the activation of pancreatic islet hormones, and hepatic glucose transporters (Glut1 and 2) [150]. Melatonin attenuates the glucose-mediated release of insulin from pancreatic cells [151]. The reduction in melatonin secretion induced by nocturnal light exposure is a crucial factor for T2DM development [136, 152, 153]. Furthermore, the melatonin receptor 1 (MT1) is involved in the regulation of glucose homeostasis and stimulates the secretion of insulin to induce glucose uptake [43]. In humans, melatonin administration attenuates glucose tolerance and insulin resistance [44]. Melatonin could suppress mitochondrial dysfunction against insulin resistance in Male Zucker diabetic fatty rats [154]. Furthermore, melatonin attenuates the secretion of pro inflammatory cytokines such as interleukin-6 (IL-6), tumor necrosis factor (TNF)-α, interferon (IFN)-gamma under insulin resistance condition in high fat diet mouse [155]. Melatonin is associated with metabolic pathways involved with the insulin pathway [156–158]. The phosphorylation of IRS-1, leading to the activation of phosphoinositide 3-kinase (PI-3 K), and SHP-2 protein was increased by melatonin [159, 160]. In the AD brain, the disturbance of insulin signaling is linked to the senile plaques formation [80, 161]. An impaired insulin receptor signaling triggers the decrease of insulin-mediated activation of PI-3 K/Akt signaling, resulting in hyperactivity of GSK-3 that induces tau hyperphosphorylation and Aβ accumulation [162]. The administration of melatonin rescues insulin receptor mechanisms and increases the activity of PI-3 K/Akt signaling and less Aβ accumulation and less tau hyperphosphorylation [163] (Fig. 3). One study suggested that the lack of melatonin by pinealectomy reduced insulin sensitivity [164]. The reduction of insulin levels in T1DM are linked to high melatonin levels in plasma [165]. Taking these results together, melatonin appears to be involved in the genesis of diabetes [42] accompanied by insulin resistance and high glucose, and may influence the cognitive dysfunction in diabetes-induced AD (Fig. 4). In this sense, melatonin may be a key molecule in the pathogenesis of Type 3 diabetes.

#### Melatonin protects the BBB against hyperglycemia

**Fig. 5:**
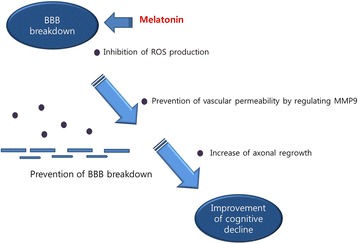
Melatonin improves cognitive decline by inhibiting BBB breakdown. Melatonin inhibits the production of ROS and the vascular permeability and increases axonal regrowth. Melatonin suppresses BBB breakdown and enhances cognitive decline

Several studies have shown that disruption of the BBB is strongly associated with cognitive dysfunction in AD [166, 167]. The BBB is a heterogeneous structure that consists of various cells important for transferring nutrients and oxygen into brain, and disruption of the BBB has been observed in patients with T2DM [168, 169]. Increases in glucose levels in the blood leads to impaired neurovascular coupling [170, 171], and increased vascular permeability [172]. Hyperglycemia-induced increases in BBB permeability lead to cognitive decline and the development of AD [101]. Hyperglycemia-induced ROS results in BBB disruption and triggers cognitive decline [101]. Dysfunction of metabolic pathways, owing to BBB disruption in diabetes, leads to cognitive deficits [173, 174]. In an in vivo study, STZ-induced diabetes results in increased BBB permeability [101]. Impaired BBB function in diabetes may be a strong risk factor for the development of AD [175, 176]. The excessive generation of ROS in T2DM has been shown to increase BBB permeability by changing tight junction protein expression [177, 178]. According to recent studies, melatonin protects BBB integrity in brain microvascular endothelial cells against inflammation [179], and protects against cerebral endothelial cell dysfunction via nicotinamide adenine dinucleotide phosphate (NADPH) oxidase-2 [180]. Moreover, melatonin prevents the increase in BBB permeability by inhibiting matrix metalloproteinase-9 expression [41]. In addition, melatonin protects against the loss of tight junction proteins and BBB disruption by promoting anti-inflammatory and antioxidant mediators, and axonal regrowth [29]. Melatonin reduces the oxidative stress-induced generation of ROS in brain endothelial cells [181], and ameliorates BBB permeability and nitric oxide levels caused by oxidative stress [182, 183]. In addition, melatonin protects against the degradation of tight junction proteins, BBB disruption, serves as an anti-inflammatory and angiogenesis regulator, and promotes axonal regrowth under high glucose conditions [29, 184]. Based on previous reports, melatonin might alleviate BBB breakdown in diabetes-induced AD by inhibiting the loss of tight junction and the increase of BBB permeability (Fig. 5).

### Conclusions and prospects

Diabetes-induced AD has been called “type 3 diabetes” owing to the common risk factors, which include insulin resistance and hyperglycemia. Here, we reviewed the effect of melatonin in type 3 diabetes from various angles. Melatonin influences type 3 diabetes by 1) suppressing Aβ toxicity and tau hyperphosphorylation, 2) controlling insulin resistance and hyperglycemia, and 3) preventing hyperglycemia-induced BBB disruption. Hence, we suggest that melatonin would be a key in attenuating the pathogenesis of type 3 diabetes.

## Abbreviations

AD: Alzheimer’s disease
Aβ: Beta amyloid
BBB: Blood brain barrier
CNS: Central nervous system
CSF: Cerebrospinal fluid
GLUT: Glucose transporter
Glut1: Glucose transporter 1
GSK-3: Glycogen synthase kinase
HIF-1α: Hypoxia-inducible factor-1α
IFN-γ: interferon –gamma
IL-6: interleukin-6
IRs: Insulin receptors
MCI: Mild cognitive impairment
MT1: Melatonin receptor 1
NFT: Intracellular neurofibrillary tangles
PI3K: Phosphoinositol-3-kinase
ROS: Reactive oxygen species
STZ: Streptozotocin
T1DM: Type 1 diabetes
T2DM: Type 2 diabetes
TNF-α: tumor necrosis factor-α
VEGF: Vascular endothelial growth factor
