# Correction to: The role of melatonin in the onset and progression of type 3 diabetes

**DOI:** 10.1186/s13041-017-0333-8

**Published:** 2017-12-08

**Authors:** Juhyun Song, Daniel J. Whitcomb, Byeong C. Kim

**Affiliations:** 1 Department of Biomedical Sciences, Center for Creative Biomedical Scientists at Chonnam National University, Gwangju, 61469 South Korea; 2 Henry Wellcome Laboratories for Integrative Neuroscience and Endocrinology, School of Clinical Sciences, Faculty of Healthy Sciences, University of Bristol, Whitson street, Bristol, BS1 3NY UK; 3 Department of Neurology, Chonnam National University Medical School, Gwangju, 61469 South Korea

## Correction to: Molecular Brain (2017) 10:35 DOI: 10.1186/s13041-017-0315-x

In the original version of this article [1], published on 1 August 2017, Fig. 3 contains a typo. In this Correction the incorrect and correct version of Fig. 3 are shown.

- Figure 3 was originally published like this:
- The correct version of Fig. 3 looks like this:

**Fig. 3:**
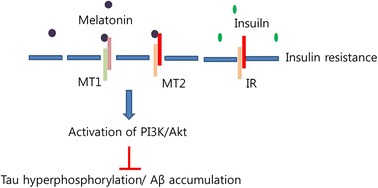
Melatonin restores the disruption of insulin signaling in AD. In insulin resistance condition, melatonin activates PI3K/Akt signaling, leading to the decrease of tau hyperphosphorylation and Aβ accumulation

**Fig. 3:**
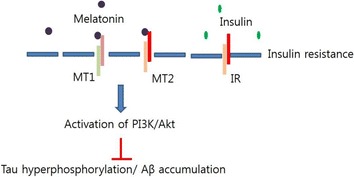
Melatonin restores the disruption of insulin signaling in AD. In insulin resistance condition, melatonin activates PI3K/Akt signaling, leading to the decrease of tau hyperphosphorylation and Aβ accumulation
